# Effectiveness of the platelet-rich fibrin in the control of pain associated with alveolar osteitis: a scoping review

**DOI:** 10.1007/s00784-023-05012-3

**Published:** 2023-04-04

**Authors:** Giusy Rita Maria La Rosa, Antonia Marcianò, Carlotta Ylenia Priolo, Matteo Peditto, Eugenio Pedullà, Alberto Bianchi

**Affiliations:** 1 Department of General Surgery and Medical-Surgical Specialties, University of Catania, Catania, Italy; 2 Department of Clinical and Experimental Medicine, University of Messina, Messina, Italy; 3 Postgraduate School of Oral Surgery, Department of Biomedical, Dental Sciences and Morphofunctional Imaging, University of Messina, Messina, Italy

**Keywords:** Alveolar osteitis, Dry socket, Pain control, Platelet-rich fibrin, Scoping review

## Abstract

**Abstract:**

**Objectives:**

The aim of this scoping review was to determine the effectiveness of the platelet-rich fibrin in the control of pain associated with alveolar osteitis.

**Materials and methods:**

Reporting was based on Preferred Reporting Items for Systematic Reviews and Meta-Analyses (PRISMA) Extension for Scoping Reviews. A literature search was conducted in the PubMed and Scopus databases to identify all clinical studies on the application of platelet-rich fibrin in the control of pain caused by alveolar osteitis. Data were extracted independently by two reviewers and qualitatively described.

**Results:**

The initial search returned 81 articles, with 49 identified after duplicates removal; of these, 8 were selected according to the inclusion criteria. Three of the eight studies were randomized controlled clinical trials, and four were non-randomized clinical studies, two of which were controlled. One study was case series. In all of these studies, pain control was evaluated using the visual analog scale. Overall, the use of platelet-rich fibrin resulted effective in the control of pain determined by alveolar osteitis.

**Conclusions:**

Within the limits of this scoping review, the application of platelet-rich fibrin in the post-extra-extraction alveolus reduced the pain associated with alveolar osteitis in almost all the included studies. Nevertheless, high-quality randomized trials with adequate sample size are warranted to draw firm conclusions.

**Clinical relevance:**

Pain associated with alveolar osteitis causes discomfort to the patient and is challenging to be treated. Use of platelet-rich fibrin could be a promising clinical strategy for pain control in alveolar osteitis if its effectiveness will be confirmed by further high-quality studies.

## Introduction

Alveolar osteitis (AO) or “dry socket” is a widely recognized complication of dental extraction caused by a partial or total disintegrated blood clot within the extraction socket. Dry socket results in inflammation of exposed alveolar bone and delayed healing, accompanied by gradually increasing severity of pain which may radiate to the auricular and temporal regions [1]. The incidence of AO ranges between 1 and 30%, being more frequent in female patients after mandibular third molar extraction [2]. Many predisposing factors have been identified for the occurrence of this phenomenon including preexisting systemic diseases, drug and oral contraceptives assumption, operative techniques, and hygiene habits [1, 3, 4]. Strong halitosis, foul taste, edema of gingival tissues with local lymphadenitis, and pain are frequent. Specifically, the severe, throbbing, referred pain is one of the most typical clinical manifestations [5]. Generally, pain associated with tooth extraction resolves in a few days by analgesics; when it persists for more days, it could be an indicator of the AO [5].

Since pain is the main and debilitating symptom of this pathology, several strategies have been proposed in order to avoid or reduce the pain associated with alveolar osteitis. The main therapeutic approaches include alveolar lavage, chlorhexidine mouthwash, application of topical gels, analgesics, cryotherapy, antibiotics, topical anesthetics and obtundent, or their combination, and placement of medicated dressings [6–10]. Therapeutic alternatives are numerous, heterogeneous, and challenging to compare [11].

Platelet-rich fibrin (PRF) is a second-generation platelet concentrate produced without biochemical blood manipulation [12, 13]. It is constituted of three key elements: first, the platelets and their activated growth factors [14]; second, the leucocytes and their cytokines [15, 16]; third, the density and complex organization of the fibrin matrix architecture produced by a natural polymerization [14]. The fibrin matrix seems responsible for the slow release of growth factors during the proliferation stage of wound healing and serves as a scaffold for cell migration and differentiation [17]. PRF is an important reservoir of numerous growth factors to promote angiogenesis, such as transforming growth factor b (TGF-b) and vascular endothelial growth factor (VEGF) [17]. In addition, PRF was found to reduce pain, swelling, and alveolar osteitis’ occurrence, as well as improve soft and hard tissue healing after mandibular extractions by a stimulation of angiogenesis and increase of local perfusion during the healing process [18, 19]. A modified form of PRF, called advanced PRF (A-PRF), was proposed. Because of its lower speed of centrifugation, A-PRF possesses a major number of platelets and growth factors with improvement in mechanical properties compared to the traditional leukocyte-PRF (L-PRF) [20].

Despite the benefits describing, some studies reported no significantly advantage in control of pain associated with AO when PRF was applied [21, 22].

This scoping review aimed to determine the effectiveness of the PRF in control of pain associated with alveolar osteitis in order to provide an updated overview of the current knowledge and address the future research.

## Materials and methods

This scoping review was reported according to the Preferred Reporting Items for Systematic Reviews and Meta-Analyses (PRISMA) Extension for Scoping Reviews [23] and focused on the following research question: “What is the effectiveness of the PRF in control of pain associated with alveolar osteitis?”

### Search strategy

A literature search was conducted in the PubMed and Scopus databases on 18/11/2022 to identify all pertinent studies investigating the effectiveness of the PRF in control of pain caused by alveolar osteitis. The following keywords were adopted for each database: (“alveolar osteitis” OR “dry socket”) AND (“platelet rich fibrin” OR “PRF”). No language restriction was used. Reference lists of selected studies were further screened for other relevant studies. Principal peer-reviewed scientific journals in oral surgery and miscellaneous (*International Journal of Oral and Maxillofacial Surgery*, *Oral Surgery Oral Medicine Oral Pathology Oral Radiology*, *Journal of Stomatology*, Oral and Maxillofacial Surgery, *Journal of Oral and Maxillofacial Surgery*, *BMC Oral Health*, *Clinical Oral Investigations*, *Odontology*) were also hand searched. Two authors independently reviewed and decided which studies had to be included. Disagreement was solved through discussion or by the decision of a third expert reviewer.

### Eligibility criteria

All clinical studies (cohort studies, randomized clinical trials (RCTs), quasi-experimental studies, case report, and case series) investigating the effectiveness of PRF in pain control associated with alveolar osteitis were included. The exclusion criteria regarded the study design (in vitro and ex vivo studies, animal studies), article type (editorials, commentaries, short communication, and reviews), peer-revision (abstracts and preprint articles), and language (studies without an English abstract).

### Data extraction

For each study, the following items:

•Author (year)

•Study design

•Participants (*n*), exclusion criteria

•Socket anatomy

•Criteria for AO diagnosis

•Intervention

•Control

•Pain measure

•Follow-up

•Main findings

were tabulated. Data were extracted independently by two reviewers. Any discrepancies were solved by discussion or intervention of a third reviewer.

## Results

**Fig. 1:**
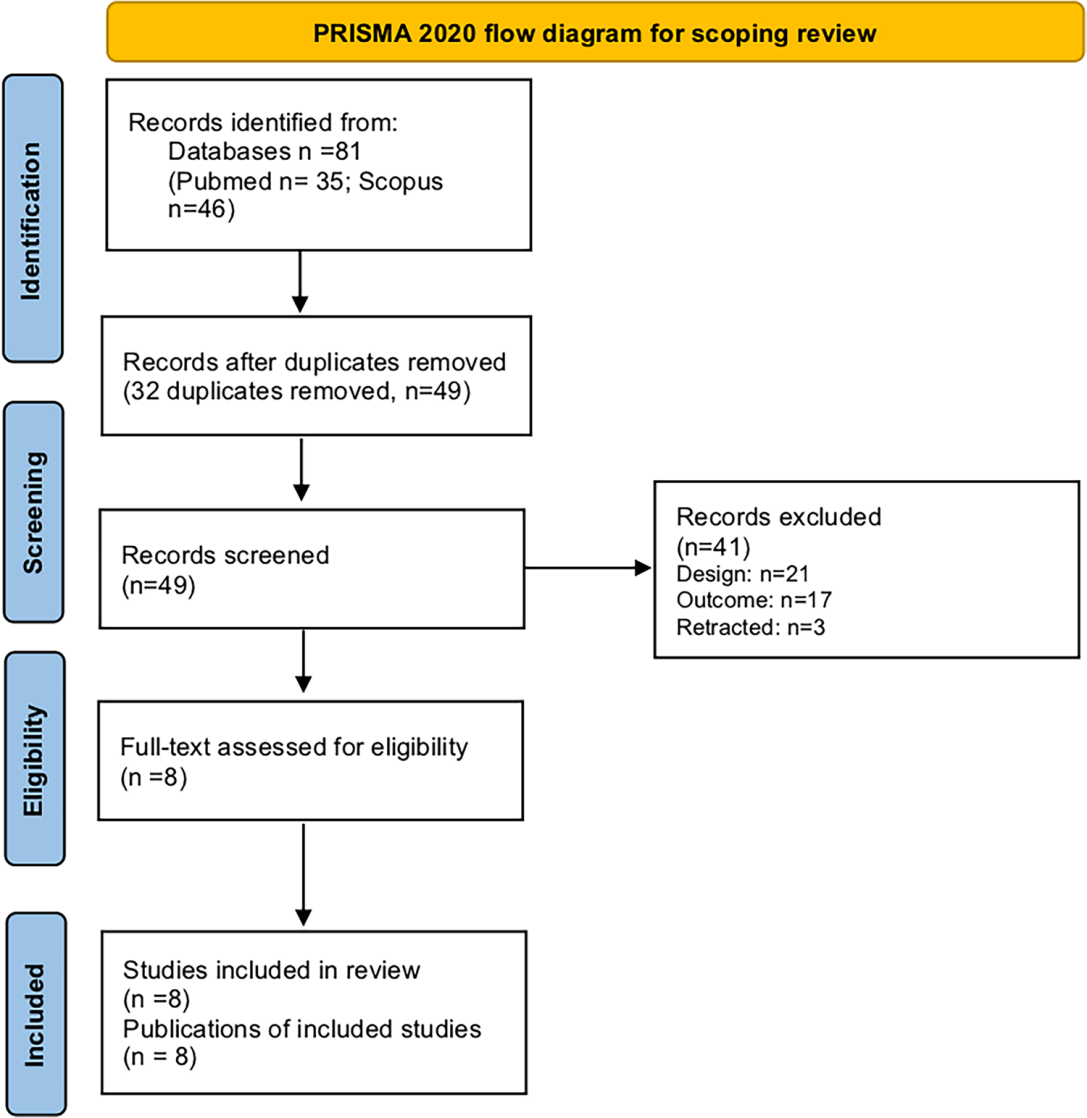
Flow chart of the review process

The electronic search resulted in 81 articles. After duplicates exclusion, 49 abstracts were reviewed, and the full texts of 8 studies were screened. Finally, 8 studies were included for qualitative analysis (Fig. 1). All included studies are listed in the Table 1.

**Table 1:** Main characteristics of included studies for the outcome of pain control

Author (year)	Study design	Participants (*n*) Exclusion criteria	Socket anatomy	Criteria for AO diagnosis	Intervention	Control	Pain measure	Follow-up	Main findings
Chakravarthi et al. (2017) [18]	Case series	10: 5 males and 5 females, aged from 41 to 64 years. Patients classified in ASN category II, III, and IV, and pregnant women.	Molar region	Minimum of two symptoms and one sign.	PRF	NR	VAS, analgesic tablet.	1-, 2-, 3-, 7-, and 14-day after intervention.	Pain was lower on the 1-day in all patients with reduced analgesic use. During the follow-up, a further decrease in pain was recorded.
Chybicki and Janas-Naze (2022) [21]	Split-mouth, single-center, non-randomized controlled study.	30: 18 males, 12 females, aged from 18 to 64 years. Patients with systemic diseases or platelets’ disorders, allergic to acetylsalicylic acid, pregnant and/or lactating women, taking any drugs affecting the platelets’ function and antibiotic or anti-inflammatory drugs or any therapy for dry socket.	NR	NR	PRF	Aspirin cone	VAS	24 h and 48 h after intervention.	PRF provided better pain relief than aspirin cones in alveolar osteitis.
Hussain et al. (2018) [22]	Prospective, randomised controlled clinical study.	40 (*n* = 20 per group): 9 males, 31 females aged from 20 to 60 years. Patients with systemic diseases or compromised immunity, smokers, diabetics, and patients on steroid therapy, pregnant, and lactating women.	NR	NR	PRF	ZOE	VAS	1-, 3-, 5-, and 7-day after intervention.	The ZOE group showed faster and better pain remission at 1-, 3-, and 5-day. Yet, no significant difference emerged between the two groups at 7-day.
Keshini et al. (2020) [25]	Randomized, controlled, clinical study.	30 (*n* = 15 per group) aged 14–60 years Sex: NR. NR	NR	NR	PRF	Alvogyl (eugenol+butamben+iodoform)	VAS	1-, 3-, and 10- days after intervention.	A significant decrease in pain was recorded in both the groups at the 3-day, with no significant differences between them.
Rastogi et al. (2018) [26]	Non-randomized, observational, clinical, and prospective study.	100: 21 males, 79 females, aged from 18 to 40 years. Pregnant and lactating women or on oral contraceptives, patients with a previous history of local or antibiotic and anti-inflammatory therapy for dry socket, with any underlying systemic disease or compromised immunity.	Mandibular and maxillar molar extraction sites.	NR	PRF	NR	VAS	1-, 3-, 7-, and 14-day after intervention.	A significant reduction in pain associated with AO was reported at 3- and 7-day.
Reeshma et al. (2021) [27]	Single-blinded, non-randomized, controlled, and prospective study.	70 (*n* = 35 per group): 17 males, 53 females aged from 30 to 60. Patients with known systemic illness, immunocompromised, smokers, with poor glycemic control, on steroid therapy, and pregnant and lactating women or taking oral contraceptives. Patients undergoing third molar extractions.	NR	Intense, radiating, throbbing pain tending to increase in severity for a period between 1- and 3-day post-surgery. Extraction socket denuded the blood clot with or without halitosis.	PRF	ZOE	VAS	1-, 3-, 5-, and 7-day after intervention.	The pain intensity was lower in the PRF group than in the ZOE at all follow-up days.
Sharma et al. (2017) [28]	Single-arm, non-randomized clinical trial.	100: 21 males, 79 females aged from 18 to 40 years. Participants with any underlying systemic disease or compromised immunity or with a previous history of local or antibiotic and anti-inflammatory therapy for dry socket, pregnant, lactating women or taking oral contraceptives.	Mandibular and maxillar molar extraction site.	NR	PRF	NR	VAS	1-, 3-, 7-, and 14- day after intervention.	A significant reduction in pain associated with AO emerged at the 3- and 7- day.
Yuce et al. (2019) [24]	Prospective, randomized, controlled, clinical study.	40 (*n* = 20 per group): 18 males, 22 females aged from 18 to 40 years. Medications interfering with the healing socket process; antibiotic or anti-inflammatory drugs in the 7 days before extraction; smoking; pregnancy or lactation; menstruation; inflammation in teeth area; oral contraceptives and radiation therapy or chemotherapy in the 12 months before extraction.	Mandibular third molar.	Blum’s definition.	A-PRF^+^	Saline solution	VAS	1-, 3-, 5-, and 7-day after intervention.	The A-PRF^+^ application reported fast and continually reduced pain intensity at each time compared with the control.

*AO*, alveolar osteitis; *ASN*, American Society of Anesthesiology; *NR*, not reported; *PRF*, platelet-rich fibrin; *VAS*, visual analog scale; *L-PRF*, leukocyte–PRF; *ZOE*, zinc oxide eugenol; *A-PRF+*, advanced PRF

Symptoms for diagnosis of dry socket include continuous throbbing type of pain; radiating to ear, temple, and neck; start of pain 1–3 days post extraction; foul taste; bad breath; pain not relieved even after medication. Signs include devoid of blood clot, infected or retained roots, localized swelling, lymphadenopathy [18]

Blum’s definition for AO diagnosis: patients with increasing postoperative pain severity in and around the lower permanent third molar extraction site in 3 days after the extraction and the total or partial breakdown of the blood clot in the socket with or without halitosis [24]

All studies were published after 2015 and investigated the potential application of PRF in the management of alveolar osteitis. For our purpose, we considered only the outcome of pain control management.

Three of the eight studies were randomized controlled clinical trials [22, 24, 25], four were non-randomized clinical studies [21, 26–28], and two of which were controlled [21, 27]. One study was case series [18].

Participants were aged from 18 to 60 years [18, 21, 22, 24–28] and mostly women [22, 24, 26–28]. Patients with any underlying systemic disease or compromised immunity or pregnant/lactating women were excluded in almost all studies [18, 21, 22, 24, 26–28], as well as patients taking previous medications for dry socket [21, 26, 28], women taking oral contraceptives [24, 26–28], and smokers [22, 24, 27]. Three studies specified the clinical criteria used for AO diagnosis including continuous, radiating, throbbing pain and the onset of symptoms 1–3 days post extraction [18, 24, 27].

Among the clinical trials, no control group was reported in the studies of Rastogi et al. [26] and Sharma et al. [28]. In the other studies, the control group was represented by aspirin cone [21], saline solution [24], Alvogyl (Septodent, Inc, Wilmington, DE) [25], and zinc oxide eugenol (ZOE) [22, 27]. Yuce et al. [24] applied Advanced-RPF (A-RPF). No specification on PRF form was reported in the other studies [18, 21, 22, 25–28].

The extraction site was not specified in 4 studies [21, 22, 25, 27]; in the remaining studies, the alveolar site was the molar region area [18, 24, 26, 28].

All the studies evaluated pain control by the visual analog scale (VAS) [18, 21, 22, 24–28]. The VAS consisted of 10 units in combination with a graphic rating scale, where the leftmost score of 0 represented absence of pain and the rightmost score of 10 indicated the worst possible, unbearable, excruciating pain. Chakravarthi et al. assessed the pain relief by recording also the analgesic intake [18].

Overall, almost all the studies showed that PRF reduced the pain associated with OA and guaranteed a fast pain relief [21, 24, 26–28]. When compared with ZOE, PRF reduced the pain intensity in all follow-up days in the study of Reeshma et al*.* (2021) [27], while the PRF group showed slower and less pain remission at 1-, 3-, and 5-day in the study of Hussain et al. [22], with no difference at 7-day.

Finally, a significant decrease in pain was recorded in both the PRF and Alvogyl groups at the 3-day, with no differences between them [25].

## Discussion

Pain is considered the most frequent and uncomfortable symptom of OA that requires an effective treatment [29]. Over the years, several strategies have been proposed for the management of pain associated with alveolar osteitis [6–9]. However, no standardized protocol for treating the associated pain has been established, and choosing the best treatment option is still a challenge for clinicians. PRF is an autologous fibrin-based biomaterial entangled with platelets, leukocytes, and their cytokines. More recently, the use of applying platelet-rich fibrin in the pain control of OA has been proposed [18, 21–28].

The aim of this scoping review was to summarize the available studies on the effectiveness of platelet-rich fibrin in the pain control of OA and offer a platform for further research.

Alveolar osteitis is a complex condition which may be challenging to be clinically standardized. Criteria of dry socket according to Chakravarthi’s definition [18] include major symptoms like foul taste, bad breath, prolonged throbbing pain radiating to the ear, temple, and neck, beginning 1–3 days after the tooth extraction and not resolving after drug intake. Signs refer to lacking of a blood clot, infected or retained roots, local swelling, and lymphadenopathy. A minimum of two symptoms and one sign are necessary to make a diagnosis of alveolar osteitis. Similar is Blum’s definition [6]. Only three studies explicated on the basis of which criteria the diagnosis of AO was performed [18, 24, 27].

Although three of the eight included studies were presented as randomized controlled clinical trials, the only study that specified the randomization procedure was that of Hussain et al. [22]. Similarly, no information was reported on strategies to guarantee the blinding of patients with regard to the treatment received and the operator responsible of pain assessment. Moreover, no control group was available in three of included studies [18, 26, 28], impairing the validity of the results obtained.

Gender, age, systemic condition, smoking status, extraction site, and surgical protocol are all factors able to impact the occurrence of postoperative complications including pain [30].

The included studies showed notable differences in participant selection. In almost all studies, the mean minimum age of the included population was 18 [21, 24, 26, 28], and the maximum was 60 [18, 21, 22, 25, 27]. Age might be a determinant of surgical difficulty, due to relative root and bone stiffness which leads to more traumatic surgeries [30].

A pivotal aspect of clinical trials is to guarantee a representative sample of the population with the aim of avoiding variables that may alter the study. Systemic pathologies, such as diabetes, increase the risk of postoperative infections and delay the wound healing due to the alterations in the microvascular circulation. This alteration results in a reduced inflammatory response, and this could lead to an alteration in the perception of pain in alveolar osteitis [31]. For this reason, almost all studies excluded patients with systemic or immune disorders [18, 21, 22, 24, 26–28]. In the study of Keshini et al. [25], preexisting systemic conditions were not reported as exclusion criteria. Smokers were excluded in three studies [22, 24, 27]. Smoking is a confounding factor because nicotine releases catecholamines which are responsible for vasoconstriction and tissue ischemia [21]. Thus, findings on smokers can be different from the general population and need caution in their interpretation.

Among the studies included, only the study of Keshini et al*.* [25] did not specify the gender. The majority of remaining studies exhibited a preponderance of women [22, 24, 26–28]. Pain associated with AO is more common in females probably due to the fact that women in childbearing age are in a continuous sinusoidal fluctuation of estrogen levels able to modify the inflammation status and thus pain perception [32, 33]. In addition, the use of oral contraceptives might raise plasma fibrinolysis and increase the risk for dry socket [30]. Nevertheless, females taking oral contraceptives are generally excluded from this kind of study [24, 26–28]. Thus, the preponderant occurrence of pain associated with AO in female patients is likely to be linked with the first mechanism [32, 33].

The study of Yuce et al. [24] tested the A-PRF. The other studies did not specify the type of PRF tested. The A-PRF exhibited a more porous structure, permitting more space for trapped platelets and immune cells and consequently a higher and more pronounced release of growth factors in comparison with L-PRF [34].

Overall, the PRF preparation was performed following the standardized and validated Choukroun’s technique [35]. This technique consists of 4 steps: blood sampling, centrifugation, fibrin clot sampling, and production of membranes, fragments, or swabs for extraction sites [35].

Four studies reported that the anatomical site was the third molar area [18, 24, 26, 28]; the others did not specify which extraction site was assessed [21, 22, 25, 27]. Of note, the molar region could require more frequently a surgical extraction. Surgical approach resulted in a 10-fold increase incidence of AO in comparison with non-surgical [36]. Indeed, the alveolar modifications caused by flap reflection and bone removal are more likely to cause AO when a surgical extraction is performed [37]. Moreover, the mandible has been reported to be more affected by AO than the maxilla probably due to more deliverance of direct tissue activators linked with bone marrow inflammation which occurred in more traumatic extractions [38].

Notable differences emerged in the sample size of included studies ranging from a minimum of 10 [18] to a maximum of 100 patients [26, 28]. A small sample size may make it challenging to assess the true effect of a treatment due to the occurrence of a type II error for which the null hypothesis is incorrectly accepted and no difference between the study groups is reported [39].

Overall, most of the included studies reported a significant reduction in OA-associated pain ensuring a fast pain relief [21, 24, 26–28]. The benefits observed in terms of pain control were probably linked with the faster wound healing promoted by PRF because of the increase in chemotaxis, angiogenesis, human osteoblast, and fibroblast proliferation, as well as differentiation in human bone mesenchymal stem cells [40, 41]. In addition, PRF favors the natural resurfacing of the dry socket wound which covers the exposed nerve terminals determining a soothing effect [28]. Moreover, the growth factors antagonize the inflammatory kinins released from the dry socket promoting the pain relief [18]. Thus, PRF could be considered an adequate healing biomaterial for pain management [17]. PRF reduced pain less than the ZOE group [22] at 1-, 3-, and 5-day post-intervention and with no significant differences compared with Alvogyl [25]. Alvogyl is an intraalveolar dressing material, largely used in the management of dry socket because it quickly provides pain relief and soothing effect during the healing process [25]. This mechanism is imputable to the analgesic, anesthetic, and antimicrobial effects of eugenol, butamben, and iodoform, respectively [25].

Interestingly, PRF was compared with ZOE in OA-associated pain remission, with contrasting results [22, 27]. ZOE is a commonly used obtundent material with antibacterial properties [22]. The different outcomes at 1-, 3- and 5-day post-intervention is probably due to the differences in sample size, enrollment population procedure, and demographical characteristics of patients involved [22, 27].

Scoping review is a flexible approach introduced for investigating the available knowledge on a specific or new topic, for determining the search boundaries, and directing the future studies [42]. The use of platelet-rich fibrin in the control of pain caused by alveolar osteitis is a current topic for which the available studies are few. Consequently, the main purpose of this scoping review was exploring and defining the applications on PRF for pain associated with alveolar osteitis as well as underlining the limitations of the current research. For this reason, all available clinical studies were included independently from study design and quality conduct. Furthermore, the bias assessment of studies was not performed being beyond the purpose of scoping review and resulting more preferable for a systematic revision approach.

Some limitations have to be considered. First, the great variety in methodology of included studies made difficult a comparison among them. In addition, pain is a subjective experience, which means that it cannot be directly verified by those who are not experiencing it. This subjectivity generates a bias that is difficult to correct, since it is mainly due to the past experiences of individuals that can affect individual pain perception [43]. The application of dressing materials inside the extraction socket has been reported to delay wound healing and cause adverse reactions [7]; however, most of the studies reporting these findings are obsolete and thus poorly informative [44–46].

Although the application of platelet-rich fibrin in the post-extraction socket is a time-consuming and invasive technique [22], it might be a promising strategy for the control of pain associated with alveolar osteitis being biocompatible, effective, and safe treatment [18, 47]. Yet, high-quality randomized clinical trials on large sample size with adequate control groups are extremely warranted to evaluate the true benefits of the application of PRF in pain associated with alveolar osteitis.

## Conclusions

Within the limits of the present scoping review, the application of platelet-rich fibrin in the post-extra-extraction alveolus reduced the pain associated with alveolar osteitis in almost all the included studies. Yet, high-quality randomized trials with adequate sample size are necessary to corroborate these findings.
